# Supplementary material for: MetaRibo-Seq measures translation in microbiomes
Source: Nat Commun. 2020 Jun 29;11:3268. doi: 10.1038/s41467-020-17081-z (PMC7324362; doi:10.1038/s41467-020-17081-z)
Supplement: Supplementary file 10 — Supplementary Data 7 [file 41467_2020_17081_MOESM10_ESM.zip › File2/Confidence_High_Taxonomy/57010_out.krona.html]

Javascript must be enabled to view this page.

members
magnitude
magnitudeUnassigned
count
unassigned
taxon
rank

57010\_out

27

2
superkingdom
27

phylum
1239
27

27
class
186801

order
186802
27

family
541000
27

genus
216851
27

853

SRS011084\_contig\_number\_4996SRS011134\_contig\_number\_25667SRS014235\_contig\_number\_contig-100\_5558.97135SRS014948\_contig\_number\_contig-100\_9143.95703SRS015264\_contig\_number\_16415SRS016335\_contig\_number\_contig-100\_8356.215177SRS019693\_contig\_number\_contig-100\_2521.44946SRS024435\_contig\_number\_contig-100\_18716.139043SRS045645\_contig\_number\_16824SRS046502\_contig\_number\_contig-100\_3534.80514SRS049959\_contig\_number\_contig-100\_11021.193701SRS056259\_contig\_number\_contig-100\_13765.113491SRS075078\_contig\_number\_24376SRS075773\_contig\_number\_33366SRS076929\_contig\_number\_contig-100\_1566.153569SRS098644\_contig\_number\_contig-100\_9508.203182SRS101433\_contig\_number\_contig-100\_1005.110630SRS1041037\_contig\_number\_contig-100\_1988.84237SRS1041038\_contig\_number\_contig-100\_1732.38092SRS105082\_contig\_number\_15829SRS140492\_contig\_number\_27615SRS142980\_contig\_number\_3276SRS143148\_contig\_number\_20121SRS147977\_contig\_number\_5379SRS149181\_contig\_number\_6927SRS149784\_contig\_number\_26040SRS893327\_contig\_number\_contig-100\_6229.40116
species
27
